# The association between coronary microvascular dysfunction and HbA1c in patients with unstable angina pectoris

**DOI:** 10.3389/fcvm.2026.1582396

**Published:** 2026-03-23

**Authors:** Kangming Li, Jing Wang, Shuang Liu, Chunmei Qi

**Affiliations:** 1 Department of Emergency, Guiling People's Hospital, Guilin, China; 2 Department of Cardiology, The Second Affiliated Hospital of Xuzhou Medical University, Xuzhou, China; 3 Xuzhou Medical College, Xuzhou, Jiangsu, China

**Keywords:** coronary microvascular dysfunction, angiographic microvascular resistance, glycemic metabolism, HbA1c, unstable angina pectoris

## Abstract

**Background:**

Angiographic microvascular resistance (AMR) is an effective parameter for assessing coronary microcirculatory function, and studies have indicated its association with adverse cardiovascular outcomes. However, the relationship between glycemic metabolic status and AMR levels requires further investigation.

**Methods:**

A total of 291 patients with unstable angina who underwent percutaneous coronary intervention (PCI) in the Cardiology Department of the Second Affiliated Hospital of Xuzhou Medical University from December 2022 to December 2023 were consecutively included, Based on the status of coronary microcirculation function, the enrolled patients were divided into a non-coronary microcirculation dysfunction group (*n* = 172) and a coronary microcirculation dysfunction group (*n* = 119). The baseline characteristics and correlation between glucose metabolism levels and microcirculation dysfunction were compared between the two groups.

**Results:**

Compared to the non-coronary microcirculation dysfunction group, the coronary microcirculation dysfunction group had a higher proportion of female patients and higher levels HbA1c (*p* < 0.05). Among all patients, univariate logistic regression analysis showed that higher HbA1c levels were significantly associated with an increased risk of coronary microcirculation dysfunction (OR = 2.037, 95% CI: 1.588–2.614, *p* < 0.005). After multivariate adjustment, elevated HbA1c levels remained an independent risk factor for coronary microcirculation dysfunction in patients with unstable angina pectoris undergoing PCI (OR = 2.061, 95% CI: 1.598–2.657, *p* < 0.001).

**Conclusions:**

The HbA1c level is independently linked to a higher risk of post-PCI CMD.

## Introduction

Acute Coronary Syndrome (ACS) is a leading cause of global cardiovascular mortality, with unstable angina (UA), a significant ACS subtype, often preceding acute myocardial infarction (1). Early intervention in this phase has been shown to reduce cardiovascular mortality by 31% (2). However, some patients with unstable angina continue to experience myocardial ischemic symptoms and objective ischemic evidence, even after the resolution of obstructive coronary lesions (3). This has led to the recognition of coronary microvascular dysfunction (CMD), a condition characterized by impaired microvascular dilation and reduced myocardial perfusion, despite the absence of significant coronary artery obstruction (4). CMD has often been underappreciated in clinical practice due to the limitations of invasive diagnostic methods. However, with advancements in non-invasive technologies such as quantitative flow ratio (QFR) and angiographic microvascular resistance (AMR), the challenges associated with CMD have gained increased attention from clinicians (5).

Despite advancements in coronary artery blood flow reconstruction techniques, cardiovascular disease mortality in China has been rising annually, contrasting with the declining trend observed in developed countries since the late 20th century (1, 2). This trend is influenced by various factors, including genetics, environmental influences, dietary habits, and population aging (6). Therefore, understanding and addressing the risk factors for unstable angina microcirculatory disorders are particularly important for the early control of coronary artery risk and prevention of long-term cardiovascular events. According to data from the International Diabetes Federation, the number of patients with diabetes in China has been increasing every year, accounting for 24% of the global diabetic population, surpassing that of any other country (7). Numerous analyses have highlighted glucose metabolism as a key cardiovascular metabolic risk factor (8–10). While HbA1c is a well-established marker of long-term blood glucose control, its relationship with coronary microcirculatory function remains underexplored (11). By exploring the association between HbA1c levels and coronary microvascular function, this study may offer new insights into the role of blood glucose control in microcirculatory protection following percutaneous coronary intervention (PCI). Additionally, it provides clinicians with a novel tool for early identification of high-risk patients, thereby facilitating the optimization of treatment strategies.

Therefore, this study aimed to apply the AMR technology to explore the association between glucose metabolism and coronary microcirculation in patients with unstable angina.

## Materials and methods

### Study population

This study enrolled patients diagnosed with UA who presented with chest pain and underwent successful PCI at the Second Affiliated Hospital of Xuzhou Medical University between December 2022 and December 2023. The main exclusion criteria were as follows: (1) age <18 years; (2) history of coronary artery bypass grafting; (3) hypothyroidism or hyperthyroidism; (4) cirrhosis, anemia, or dialysis; (5) cirrhosis, anemia, or dialysis; (6) previous myocardial infarction and cardiomyopathy; and (7) incomplete baseline data. Initially, 320 patients with unstable angina pectoris were assessed; after applying the exclusion criteria, 291 patients were included in the study.

### Data collection

Demographic data (age, sex and concomitant diseases) were obtained from medical records. Levels of glycated hemoglobin A1c (HbA1c), triglycerides, total cholesterol, high-density lipoprotein cholesterol (HDLc), low-density lipoprotein cholesterol (LDLc), and very low-density lipoprotein (VLDL) were measured immediately after admission.

AMR was assessed using the QFR software (Shanghai Biodong Medical Technology Co., Ltd., Shanghai, China). An AMR value greater than 2.5 mmHg*s/cm reflects abnormal coronary microcirculation in patients (5, 12).

AMR was assessed immediately following successful PCI of the target vessel, defined as restoration of TIMI flow grade 3 with residual stenosis <20%. The method for calculating single-view µQFR involved delineating the coronary artery lumen contour of the patient under examination using the QFR software. Congestive flow velocity was determined by dividing the length of the vessel centerline by the contrast filling time (13). Subsequently, a frame with well-filled and fully exposed lumen contours was selected and the boundaries of the vessels and major side branches were outlined. The reference vessel diameter was reconstructed based on Murray's law of bifurcation fractals, accounting for the decrease in luminal diameter at bifurcations (14). Finally, using the aforementioned hyperemic flow as the boundary condition, the pressure drop is calculated based on the fluid dynamics equation. Distal coronary pressure (Pd) was determined based on the pressure drop. µQFR was calculated as the ratio of Pd to the mean aortic pressure (Pa), whereas AMR was calculated as the ratio of Pd to the hyperemic flow velocity (V_hyp_).

$$\mathrm{AMR}\;=\;\mathrm{Pd}\;/\;V_{\mathrm{hyp}}\;=\;\mathrm{Pa}\;\;\times \;\mu \mathrm{QFR}\;/\;V_{\mathrm{hyp}}$$

### Data analysis

All statistical analyses in this study were performed after the research protocol received approval from the Ethics Committee of the Second Affiliated Hospital of Xuzhou Medical University (2020120205) and after obtaining written informed consent from all participants. Continuous variables following a normal distribution are expressed as mean ± standard deviation, while categorical variables are presented as counts (percentages). Between-group comparisons were made using t-tests for continuous data and chi-square tests for categorical data. The relationship between glycemic levels and coronary microvascular dysfunction was evaluated using univariate and multivariate logistic regression analyses. Spearman's correlation coefficient was used for non-normally distributed continuous variables. A *p*-value <0.05 was considered statistically significant. All analyses were conducted using SPSS 27.0, Grapdprism and MedCalc statistical software.

## Results

### Comparison of clinical data between two groups of patients

As shown in Table 1, compared with the non-coronary microvascular dysfunction group, the coronary microvascular dysfunction group had a lower proportion of female patients and higher HbA1c levels (*p* < 0.05).

**Table 1:** Baseline characteristics of studied patients.

Variable	N-CMD (*N* = 172)	CMD (*N* = 119)	*p*
Age	66.11 ± 11.22	66.08 ± 11.24	0.984
Female (*N*,%)	127 (73.8%)	68 (57.1%)	0.003
Smoking	50 (29.1%)	35 (29.4%)	0.950
Alcohol consumption	32 (18.6%)	20 (16.8%)	0.694
Hypertension	104 (60.5%)	73 (61.3%)	0.880
DM	35 (20.3%)	68 (51.7%)	**<0** **.** 001*
AF	10 (5.8%)	9 (7.6%)	0.553
TG	1.706 ± 1.502	1.598 ± 1.058	0.501
CHOL	4.36 ± 1.81	4.44 ± 1.24	0.584
HDLC	1.08 ± 0.28	1.11 ± 0.23	0.455
LDLC	2.41 ± 0.85	2.51 ± 0.92	0.312
VLDL	0.78 ± 0.68	0.72 ± 0.48	0.499
FBG	5.73 ± 2.18	6.78 ± 2.66	**<0** **.** 001*
HbA1c	6.14 ± 0.88	7.25 ± 1.82	**<0** **.** 001*
AMR	2.23 ± 0.23	2.78 ± 0.26	**<0** **.** 001*

DM, diabetes mellitus; AF, atrial fibrillation; TG, triglyceride; CHOL, cholesterol; HDLC, high density lipoprotein; LDLC, low density lipoprotein; VLDL, very low density lipoprotein; FBG, fasting blood glucose; HbA1c, glycated hemoglobin A1c; AMR, angiographic microvascular resistance.

Bold term and * indicates statistically significant.

### Relationship between HbA1c and coronary microvascular dysfunction in patients with unstable angina

**Figure 1:**
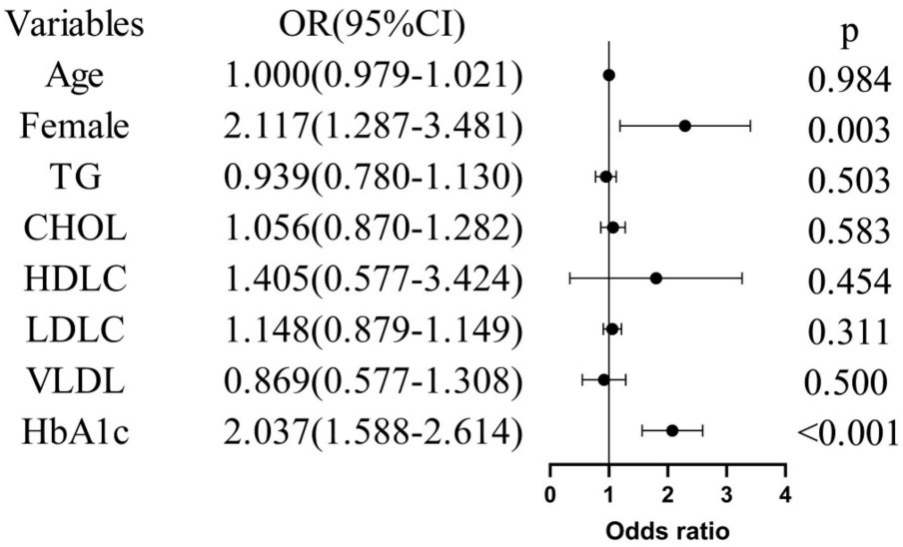
Univariate logistic regression analysis of the events. TG, triglyceride; CHOL, cholesterol; HDLC, high density lipoprotein; LDLC, low density lipoprotein; VLDL, very low density lipoprotein; FBG, fasting blood glucose; HbA1c, glycated hemoglobin A1c.

As shown in Figure 1, in the univariate logistic regression model, sex and HbA1c level were positively correlated with the risk of coronary microvascular dysfunction in patients with unstable angina after PCI (*p* < 0.05).

**Figure 2:**
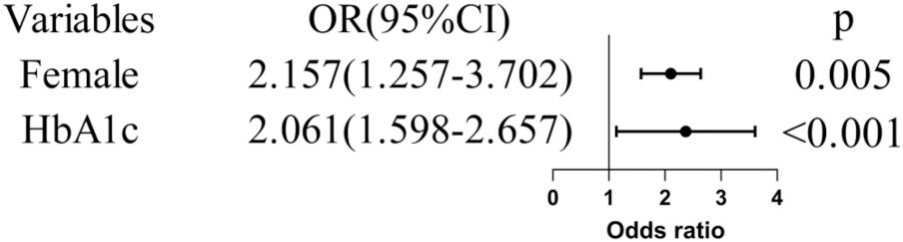
Multivariate logistic regression analysis of the events. HbA1c, glycated hemoglobin A1c.

In univariate analysis, both female sex and HbA1c levels were significantly associated with CMD risk (*P* < 0.05). After including both variables simultaneously in a multivariate logistic regression model, and adjusting for the factor of female sex, HbA1c levels remained independently and positively correlated with the risk of coronary microvascular dysfunction (Figure 2) (adjusted odds ratio [OR] = 2.061; 95% CI: 1.598–2.657, *p* < 0.001).

### Glycated hemoglobin and microvascular resistance Index

**Figure 3:**
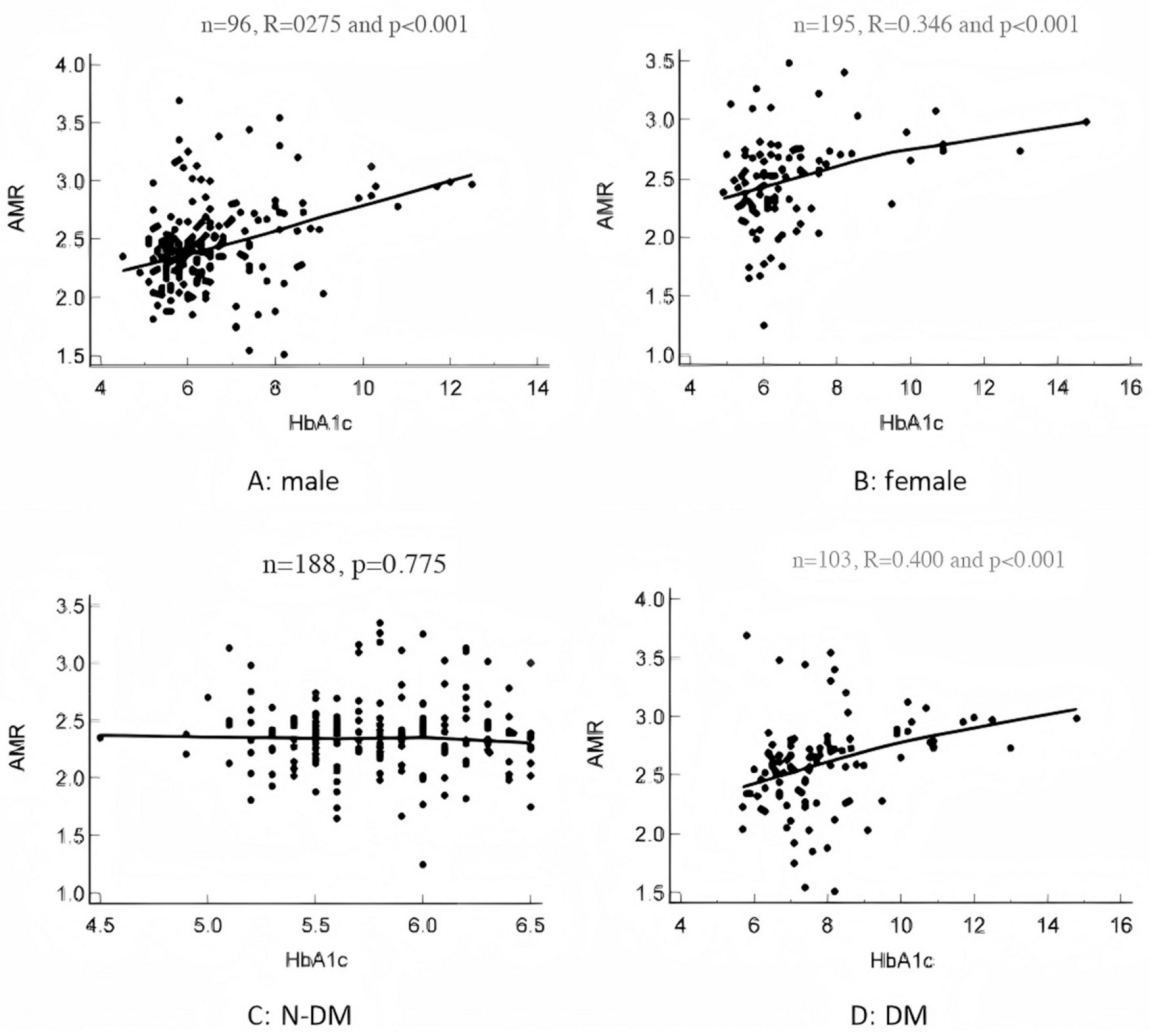
Glycated hemoglobin and microvascular resistance index. **(A)**: In male population, HBA1c was positively correlated with AMR. **(B)**: In female population, HBA1c was positively correlated with AMR. **(C)**: In the non-diabetic population, HBA1c is not associated with AMR. **(D)**: In the diabetic population, HBA1c was positively correlated with AMR. N-DM, none-diabetes mellitus; DM, diabetes mellitus; AMR, angiographic microvascular resistance.

As shown in Figure 3, glycated hemoglobin in both male and female patients was positively correlated with AMR (male: *p* < 0.001, R = 0.275; female: *p* < 0.001, R = 0.346). An additional comparison of the correlation coefficients between the two groups demonstrated no statistically significant differences (*p* = 0.534). Subsequent stratification according to the presence of diabetes among patients revealed a positive correlation between glycated hemoglobin and AMR in diabetic subjects (*p* < 0.001, R = 0.400), whereas no discernible pattern was noted among non-diabetic individuals (*p* = 0.775).

## Discussion

Allen et al. conducted a multicenter prospective study in 2022, revealing that 24% of ACS patients still had residual coronary ischemia post-coronary stent implantation (3). Furthermore, individuals in this cohort exhibited a markedly elevated likelihood of experiencing cardiovascular adverse events within one year following the procedure compared to those devoid of residual ischemia (15, 16). Coronary residual ischemia, characterized by persistent myocardial ischemia after successful PCI, is often attributed to endothelial dysfunction in the coronary microvasculature (5). In this context, the identification of objective biomarkers associated with microvascular dysfunction is of particular importance, carrying specific clinical value for diabetic patients who frequently present with atypical symptoms. For instance, myocardial ischemia in this population may manifest solely as non-classical symptoms including sleep disturbances, dyspnea, or fatigue rather than typical chest pain, frequently leading to delayed diagnosis. While traditional obstructive coronary artery disease primarily involves lipid accumulation, plaque formation, and vascular narrowing or occlusion leading to myocardial ischemia, the role of the coronary microvascular system, in conjunction with epicardial coronary arteries, in maintaining myocardial blood flow is crucial (17). Currently, AMR serves as an effective tool for assessing coronary microcirculatory function (12). Numerous studies have demonstrated a significant association between post-PCI AMR and subsequent adverse cardiovascular outcomes (16, 18, 19). Therefore, the identification of risk factors associated with decreased post-PCI AMR is clinically significant for mitigating the residual risk of coronary ischemia.

In 2022, Li Jianjun's team emphasized the critical role of glycemic metabolism and noted that, compared to high-income countries, developing nations often neglect adequate blood glucose management (6). Elevated blood glucose is a key mediator of microvascular dysfunction in prediabetes and type 2 diabetes (9). Considering the challenges associated with the regular monitoring of blood glucose fluctuations in patients, HbA1c serves as a reliable indicator of glycemic levels over a defined period (11). Particularly for diabetic patients presenting with atypical symptoms, these objective biomarkers help overcome the limitations of subjective symptom assessment, thereby providing more reliable tools for risk stratification and early intervention. The study have shown that hyperglycemia upregulates COX-2 mRNA and protein expression, leading to increased thromboxane A2 synthesis and reduced prostacyclin release (9). It also induces PKC-dependent activation of NAD(P)H oxidase in endothelial cells, thereby promoting the generation of reactive oxygen species (ROS) and subsequent endothelial cell injury (20, 21). Additionally, the FAVOR III China study (2022) also found that diabetic patients have a higher risk of major adverse cardiovascular events (MACE) post-PCI compared to non-diabetic individuals (22, 23). Consequently, elevated HbA1c levels are strongly associated with increased cardiovascular risk.

In 2023, Yong J and colleagues used cardiac magnetic resonance (CMR) imaging to explore the relationship between myocardial perfusion index and metabolic parameters in patients, they observed that individuals with lower myocardial perfusion indices had elevated fasting blood glucose and HbA1c levels (24). However, the study was limited by a small sample size of 80 cases and the high detection costs associated with CMR. Building on these findings, we utilized non-invasive, low-risk, and cost-effective AMR technology to further investigate the impact of HbA1c on coronary microcirculatory function. Our results similarly showed that patients with unstable angina and coronary microcirculation dysfunction had higher fasting blood glucose and HbA1c levels. Additionally, a greater proportion of female patients was observed in the coronary microcirculation disorder group compared to the non-disorder group. Previous studies have shown a higher prevalence of coronary microvascular dysfunction in females, likely due to smaller heart and coronary artery sizes, as well as reduced estrogen levels postmenopause, which impair vasomotor function and microvascular dilation (25–27). The Magenshan team also reported that coronary microcirculatory resistance in females was higher than that assessed by FFR and QFR techniques (28).

### Methodological consideration regarding glycemic metrics

In constructing the multivariate model, we deliberately selected HbA1c over diabetes status or fasting blood glucose as the primary glycemic variable. This decision was driven by two principal considerations. First, the core scientific objective of this study was to evaluate the independent association of chronic glycemic exposure, as a continuous variable quantified by HbA1c, with CMD. Second, HbA1c, by definition, is the cornerstone for diagnosing and managing diabetes and exhibits inherent, strong collinearity with both categorical diabetes status and acute FBG. Simultaneous inclusion of these highly correlated variables in a regression model would introduce severe multicollinearity, destabilizing effect estimates and obscuring the interpretation of each factor's unique contribution. While this approach effectively elucidates the role of HbA1c, it constitutes a key limitation of our analysis: it precludes precise delineation of whether the observed association is fully independent of, or interacts with, a formal diabetes diagnosis. Our correlation data, suggesting a stronger HbA1c-AMR link in diabetic patients, hints at a potential effect modification that future studies with larger cohorts should formally test via stratified analyses or interaction terms.

We posit that in contrast to inherent gender-related influences on microcirculation, heightened levels of blood glucose exert a more pronounced impact on endothelial cell impairment, consequently resulting in increased microcirculatory resistance. Given the frequent presentation of atypical symptoms in diabetic patients, intensified glycemic control combined with objective microcirculatory assessment tools such as AMR is imperative for achieving early diagnosis and precise risk stratification. Therefore, for patients with unstable angina, treatment strategies may need to be more individualized, particularly with a focus on enhanced blood glucose control and monitoring. In addition to routine cardiovascular risk management, early identification and intervention of glucose abnormalities, especially in patients with diabetes or hyperglycemia, may help to slow or prevent the progression of microvascular dysfunction. Furthermore, improving the assessment of coronary microcirculatory function during coronary interventions for unstable angina patients can facilitate the early use of medications aimed at improving coronary microcirculation, ultimately leading to better patient prognosis and improved clinical outcomes post-PCI.

## Limitation

This study had certain limitations. First, this was a single-center retrospective study that did not include follow-up information. Second, the sample size of this study was limited, and a large-sample, multicenter, prospective study is required to provide more comprehensive evidence. Third, while HbA1c was evaluated as an indicator of long-term glycemic control, the potential influence of glycemic variability on coronary microcirculatory dysfunction was not assessed. Fourth, regarding the statistical modeling, based on pathophysiological considerations of multicollinearity, we selected HbA1c rather than diabetes status as the glycemic indicator. While this facilitated the elucidation of the independent role of HbA1c, it also implies that this study cannot precisely distinguish to what extent the effect of HbA1c is independent of a diabetes diagnosis. Future studies with larger samples should employ pre-stratification or analytical strategies incorporating interaction terms to disentangle the relative contributions of these two closely related factors to CMD risk.

## Conclusion

This study demonstrates an association between HbA1c levels and CMD after PCI. Notably, this link was particularly strong within the subgroup of patients with diabetes, suggesting HbA1c's role may be more critical in this population.

## Data Availability

The raw data supporting the conclusions of this article will be made available by the authors, without undue reservation.
